# Image-Guided Fluorescence Endomicroscopy: From Macro- to Micro-Imaging of Radiation-Induced Pulmonary Fibrosis

**DOI:** 10.1038/s41598-017-18070-x

**Published:** 2017-12-19

**Authors:** Jessica R. Perez, Norma Ybarra, Frederic Chagnon, Monica Serban, Gabriel Pare, Olivier Lesur, Jan Seuntjens, Issam El Naqa

**Affiliations:** 1 McGill University, Biomedical Engineering, Montreal, H4A 3J1 Canada; 2 McGill University Health Center, Medical Physics, Montreal, H4A 3J1 Canada; 3 Sherbrooke University, Intensive Care Unit and Pulmonology, Sherbrooke, J1H 5N4 Canada; 4 University of Michigan, Radiation Oncology, Ann Arbor, MI 48103-4943 USA

## Abstract

Radiation-induced pulmonary fibrosis (RIPF) is a debilitating side effect of radiation therapy (RT) of several cancers including lung and breast cancers. Current clinical methods to assess and monitor RIPF involve diagnostic computed tomography (CT) imaging, which is restricted to anatomical macroscopic changes. Confocal laser endomicroscopy (CLE) or fluorescence endomicroscopy (FE) in combination with a fibrosis-targeted fluorescent probe allows to visualize RIPF in real-time at the microscopic level. However, a major limitation of FE imaging is the lack of anatomical localization of the endomicroscope within the lung. In this work, we proposed and validated the use of x-ray fluoroscopy-guidance in a rat model of RIPF to pinpoint the location of the endomicroscope during FE imaging and map it back to its anatomical location in the corresponding CT image. For varying endomicroscope positions, we observed a positive correlation between CT and FE imaging as indicated by the significant association between increased lung density on CT and the presence of fluorescent fiber structures with FE in RT cases compared to Control. Combining multimodality imaging allows visualization and quantification of molecular processes at specific locations within the injured lung. The proposed image-guided FE method can be extended to other disease models and is amenable to clinical translation for assessing and monitoring fibrotic damage.

## Introduction

Radiation-induced pulmonary fibrosis (RIPF) is a common side effect of thoracic irradiations for lung and breast cancer treatments^1^. Radiation therapy (RT) aims to treat the tumor and spare surrounding healthy tissue. However, despite great progress in RT delivery, some normal tissue around the tumor will be exposed to irradiation. RT results in cell kill and loss of function of the affected region. It is then followed by a reversible inflammatory phase with accumulation of fluid and recruitment of inflammatory cells to the site of injury. In some cases, when the inflammatory phase is not regulated properly, it persists and there is accumulation of extra-cellular matrix and subsequent formation of scar tissue^2,3^. This late phase which is thus far irreversible is called RIPF. The inflammatory phase that occurs prior to the fibrotic phase might not be visible on imaging or be symptomatic at that stage. In such subclinical inflammation where the inflammatory response is asymptomatic, it is not detectable unless measured at different time points with relevant methods. Currently, limited treatment options are available for RIPF and mainly involve supplementary oxygen and steroids to reduce inflammation^4^. RIPF is closely related to idiopathic pulmonary fibrosis and the methodology presented here could be extended to this disease and similar fibrotic diseases as well.

The exact underlying molecular mechanisms behind RIPF remain unknown and is an area of active research. Currently, to assess the extent of RIPF clinically, a chest x-ray (2-dimensional) or its 3D equivalent computed tomography (CT) scan is performed^5^. Both methods detect differences in tissue density as a basis of image contrast. As RIPF develops, the accumulation of extra-cellular matrix components (such as collagen) within the lung creates an increased lung density that can become visible with x-ray and CT imaging^6,7^. Radiological evidence of RIPF has been associated with RT dose^8^.

A known landmark of fibrosis is collagen deposition and its accumulation relates to the extent of damage present in the tissue^9^. CT imaging can therefore be used to monitor RIPF over time and help determine the efficacy of newly proposed therapies^10^. However, CT imaging is restricted to macroscopic anatomical information. It is true that many CT-based techniques can provide functional information, however their clinical use is limited. RIPF is mostly diagnosed clinically with anatomical CT imaging given the associated extra radiation risks with these technologies. A complementary imaging technique capable of detecting RIPF at the subcellular and molecular levels would be of great interest to better understand how RIPF develops and could be used to monitor disease progression and the therapeutic efficacy when testing new experimental drugs.

Advanced molecular imaging techniques have the potential to help answer such open biologically relevant questions. Magnetic resonance imaging (MRI) and positron emission tomography (PET) in combination with a fibrosis targeted probe have been used to visualize lung fibrosis pre-clinically at the molecular level^11,12^. Molecular imaging is not restricted to macroscopic imaging from outside the body. In particular, fluorescence endomicroscopy (FE) is a new promising minimally invasive imaging technique, which consists of a confocal fluorescent microscope at the tip of an endomicroscope^13^. FE is able to image accessible organs such as the lung through bronchoscopy, and provide “optical biopsies” of disease regions *in vivo* and in real time. FE is already used clinically with autofluorescence or in combination with fluorescent probes for bronchoscopy to study the progression of different lung diseases^14^. However, it has not been applied in clinical RIPF assessment yet. Here, we present a fluorescent collagen probe in combination with FE imaging to enable visualization of RIPF at the cellular level *in vivo* in a rat model.

One of the major limitations of FE imaging for clinical and pre-clinical practice is the lack of ability to localize the endomicroscope inside the organ of interest at the time of imaging. If the region of the lung in which the FE images are taken can be macroscopically identified, they can be related to a specific location or a disease region of interest within the lung. Fluoroscopy-guidance is used regularly for image-guided radiotherapy, surgical procedures or bronchoscopy, providing 2D x-ray images of the subject in real-time^15^.

In this study, we present and evaluate macroscopic fluoroscopy-guidance to localize the tip of the endomicroscope during FE imaging in a rat model of RIPF. This allows to relate FE microscopic information at a certain specific location within the lung, to its corresponding macroscopic 3D CT image for disease assessment and monitoring.

## Results

### Fluorescent collagen probe validation for FE imaging

**Figure 1:**
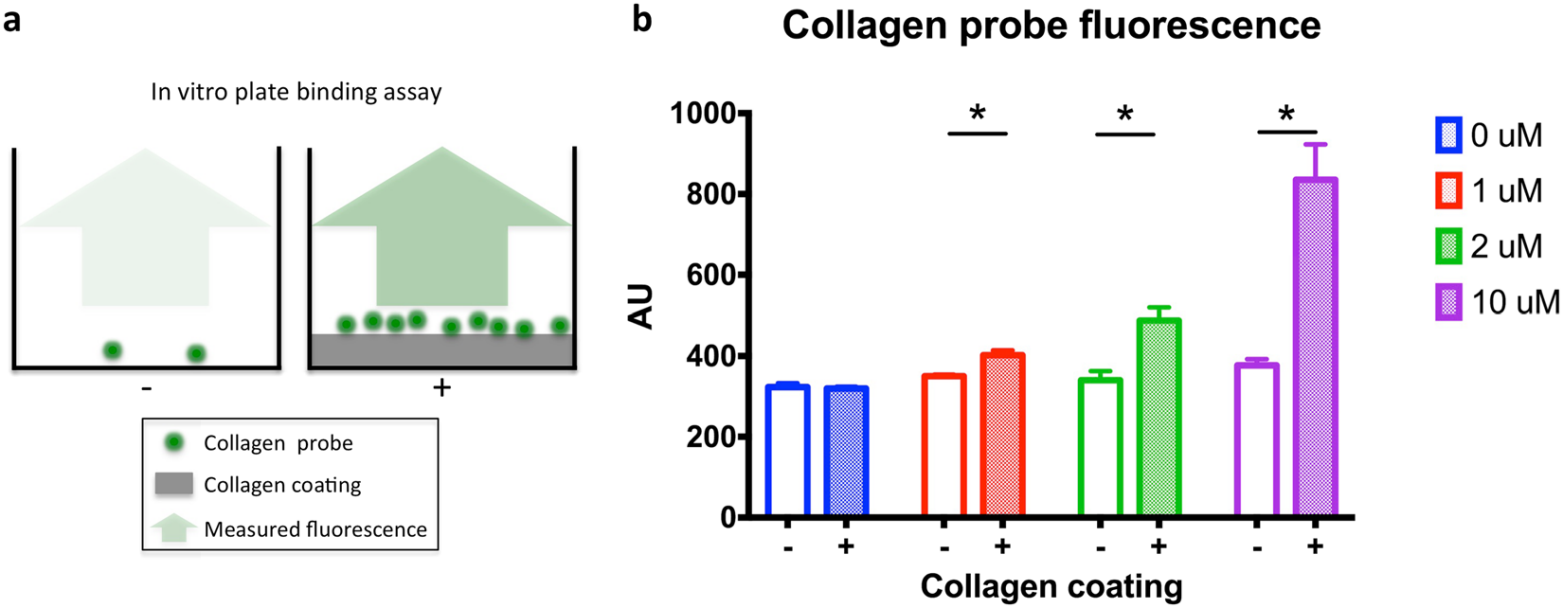
*In vitro* collagen plate binding assay. (**a**) Schematic of well plate without collagen coating (left) and with collagen coating (right). The collagen probe (green circles) binds to the collagen coating and the resulting fluorescence is measured (green arrows). (**b**) Fluorescence in arbitrary units (AU) at increasing collagen probe concentrations (from 0 *μM* to 10 *μM*) with or without collagen coating (+ or −).

In order to visualize fibrosis at the microscopic level with FE imaging, a green fluorescent collagen probe was synthesized based on the design of a collagen binding MRI probe for fibrosis imaging^16^. Prior to *in vivo* imaging, we evaluated the collagen probe binding affinity *in vitro* using a plate binding assay, where the collagen probe is added to wells of a plate that were coated (experimental) or not (control) with collagen. The measured fluorescence intensity from each well is then proportional to the collagen probe binding (Fig. 1a). An increase in fluorescence intensity in the presence of collagen coating and an increase in fluorescence with increasing collagen probe concentration were observed (Fig. 1b). A significant discrepancy in fluorescence intensity between with and without collagen coating started at 1 *μM* and increased even more at 10 *μM* (Fig. 1b, purple) (*p* = 0.0022).

Following *in vitro* validation, we tested the collagen probe *in vivo* in a bleomycin-induced lung fibrosis rat model imaged with FE. The probe injection was well tolerated and images showed an increased fluorescence in the fibrosis model compared to the control with the distinct presence of fluorescent fiber structures (data not shown).

### CT, fluoroscopy and FE imaging experimental scheme

**Figure 2:**
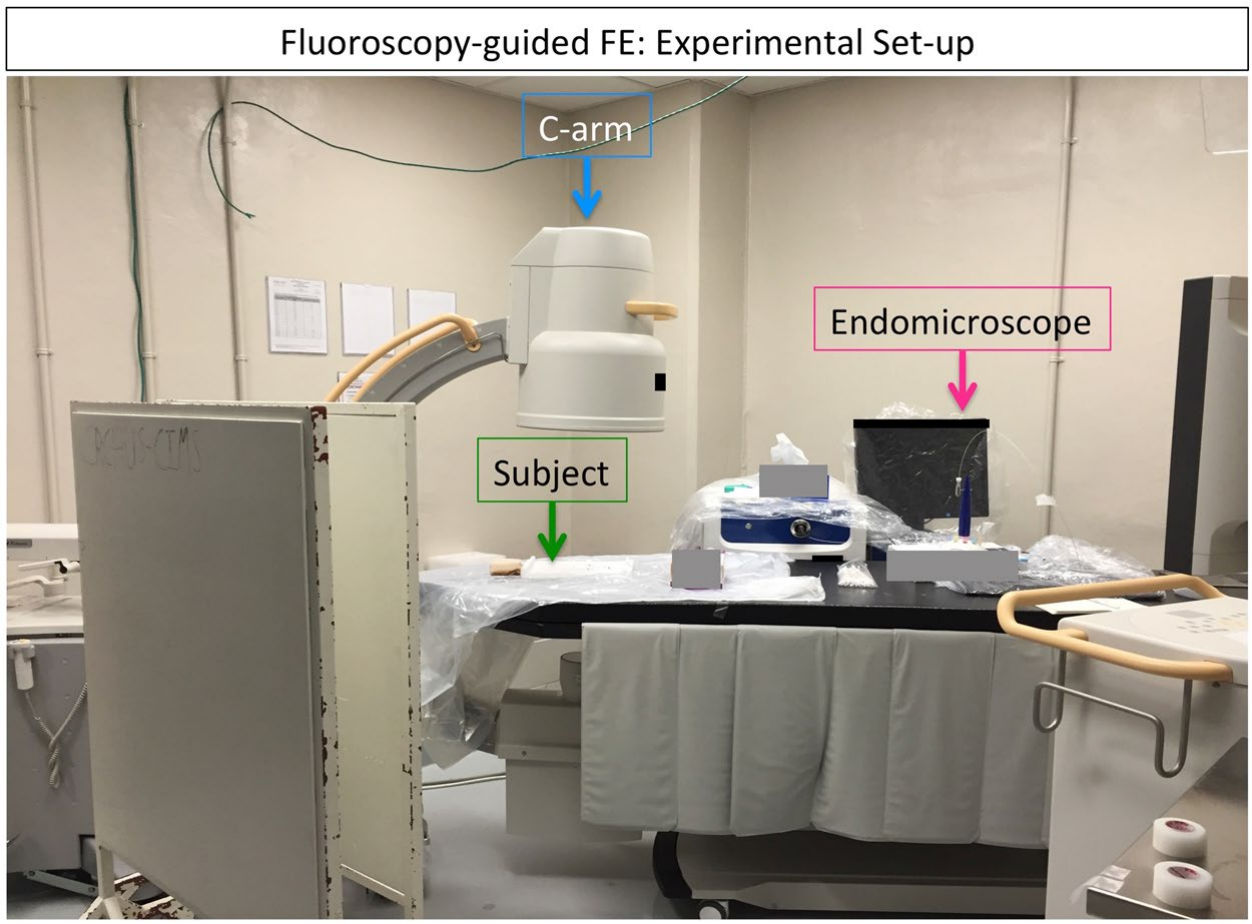
Experimental set-up. The rat is placed on the couch table (green arrow) in supine position. Then, the endomicroscope (pink arrow) is inserted through a tracheotomy to a certain position in the lungs and an FE video is acquired. With the endomicroscope in place an x-ray fluoroscopy image is acquired with the C-arm (blue arrow) in coronal view. Then, the C-arm is rotated by 90 degrees and a sagittal view image is acquired. The endomicroscope is moved to another location in the lung and the same process is repeated for every endomicroscope position.

Sprague-Dawley rats (n = 16) were divided into two groups: Control (n = 8) or receiving radiation therapy (RT) (n = 8). The RT group were treated with 18 *Gy* to the right lung (hemithorax) in order to induce pulmonary fibrosis following our previous work^17^. All rats underwent a CT scan 24 weeks following irradiation. The next day, rats were injected with the fluorescent collagen probe and imaged with FE and fluoroscopy (coronal and sagittal views) at different endomicroscope locations. The experimental setup is described in Fig. 2.

### FE images of collagen fibers

**Figure 3:**
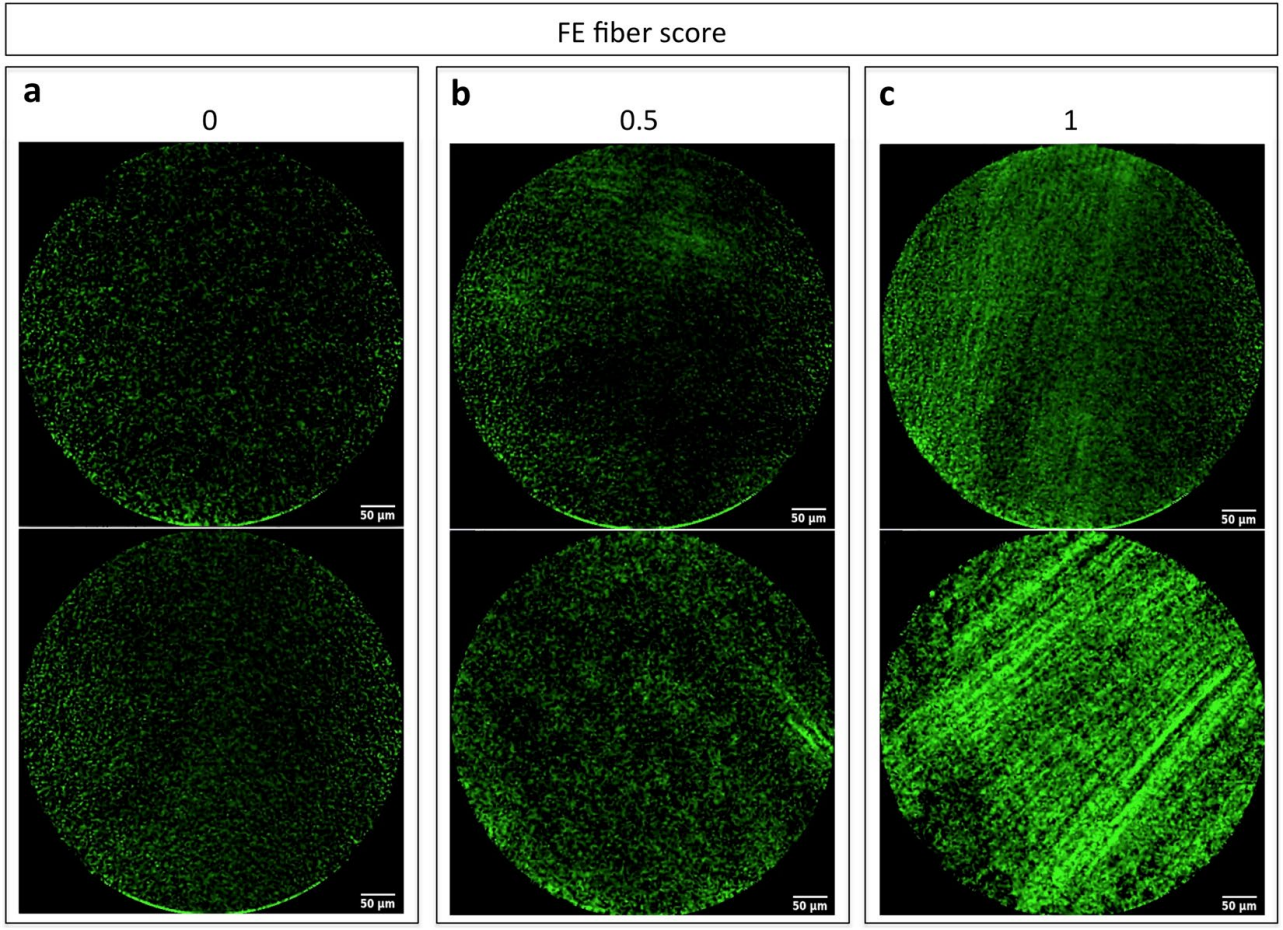
Representative FE images with collagen-targeted fluorescent probe showing fiber structures. FE video sequences were visually scored for the presence of fluorescent fibers from (**a**) 0: no fibers, (**b**) 0.5: faint appearance of fibers, to (**c**) 1: presence of fibers.

We have previously done a complete study without the collagen fluorescent probe and relying only on auto-fluorescence of the airways. As for humans, in rats, we observed fiber structures in fibrotic lungs compared to controls. However, the autofluorescent signal was quite low, so we used a targeted probe to highlight the fiber structures. FE images in combination with the fluorescent collagen probe showed the presence of fiber structures of increased fluorescence intensity as well as a noisy background of fluorescent dots. The relevant signal of interest became the detection of such fiber structures. Therefore, FE images were quantified by visual scoring of absence (0), faint appearance (0.5) or presence (1) of fibers (Fig. 3). This figure was to show the variety of fiber structures used for scoring, independent of RT status. Two independent observers scored FE video sequences for the presence of fluorescent fibers. The two observers’ scores were in good agreement with a concordance correlation coefficient (*CCC*) = 0.92, 1 being perfect agreement. The FE fiber score used subsequently for FE image quantification is the average score of the two observers.

### Image registration and endomicroscope localization

**Figure 4:**
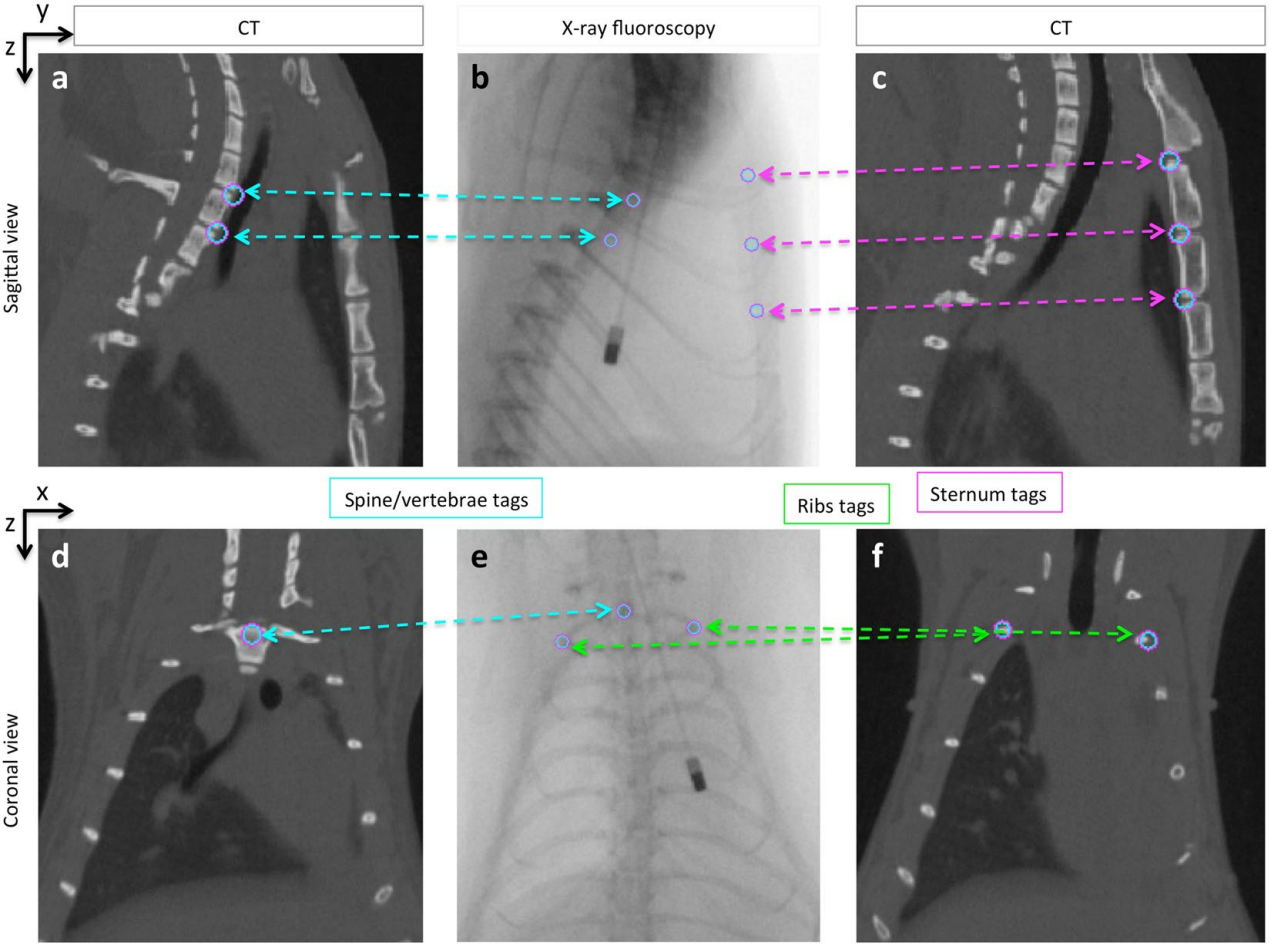
X-ray fluoroscopy to CT registration with point-matching method. Sagittal view (**a**,**b** and **c**) and coronal view (**d**,**e** and **f**) showing matching tags (circles) placed on anatomical landmarks appearing on both CT (**a**,**c**,**d** and **f**) and fluoroscopy (b and e). Tags are placed on visible structures such as the spine or vertebrae (cyan arrows), the ribs (green arrows) or the sternum (magenta arrows). The sagittal view provides the y and z coordinates and the coronal view provides the x and z coordinates.

In order to localize the endomicroscope within the lung in 3D, a pair of 2D fluoroscopy images with the endomicroscope in place (coronal and sagittal view) were registered to the CT volume. Registration of each fluoroscopy image (2D) to the corresponding CT image (3D) was performed using a point-matching method^18^. Tags were placed on the same anatomical landmarks (vertebrae, ribs and sternum) on both fluoroscopy and CT images (Fig. 4). Based on those matching points, an affine transform with 7 degrees of freedom (3 translations, 3 rotations and 1 scaling) was computed.

**Figure 5:**
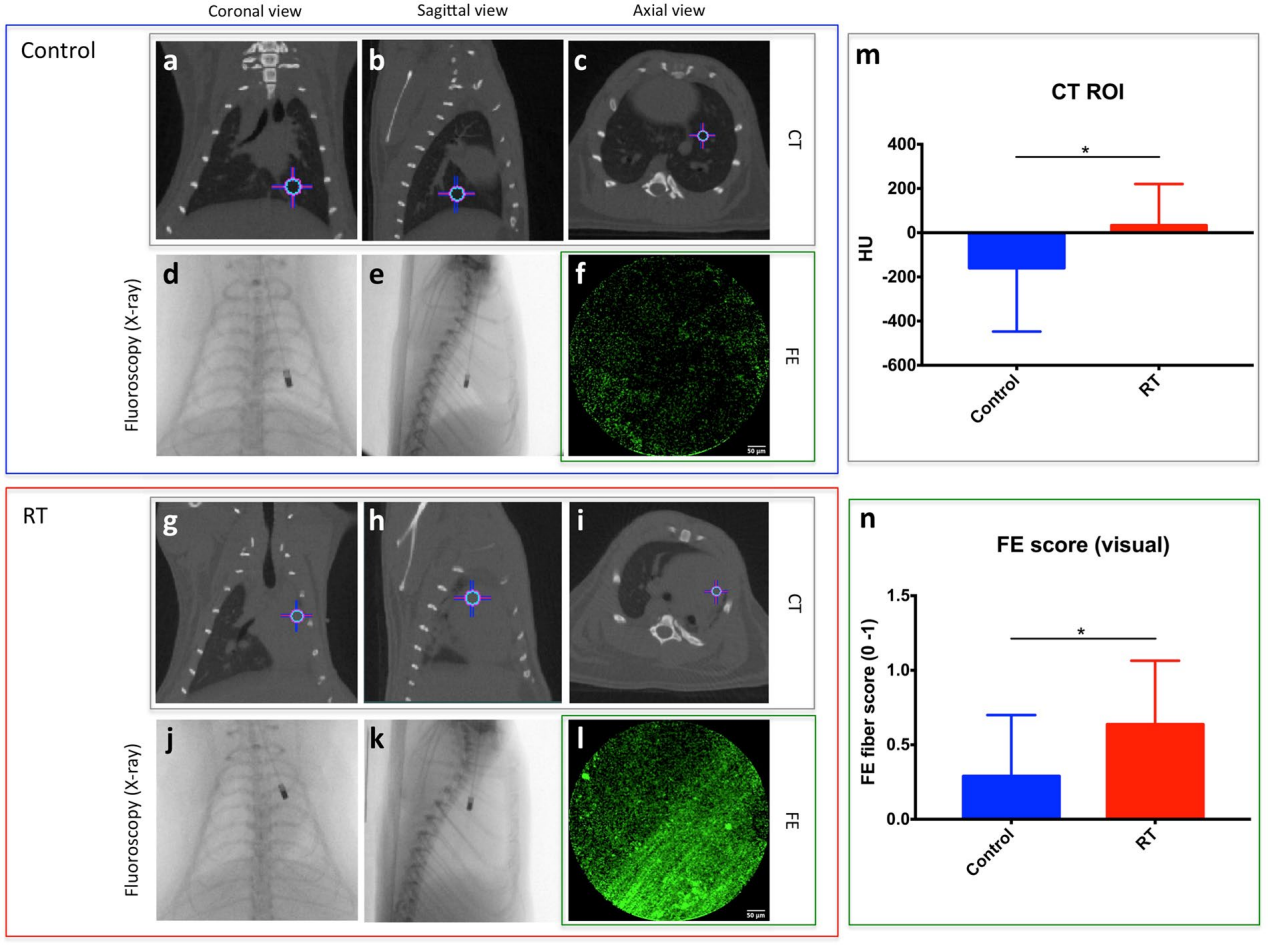
Representative CT (**a–c** and **g–i**, gray box) and FE (**f** and **l**, green box) images of control (**a**–**f**, blue box) and RT (**g–l**, red box) at the corresponding endomicroscope position determined with the fluoroscopy images (**d**,**e** and **j**,**k**, respectively). Crosshair indicates the position of the endomicroscope tip. (**m**) CT image quantification: The mean CT number (HU) for ROIs of each endomicroscope position for control (blue) and RT (red). (**n**) FE image quantification: Fiber score from 0 (no fiber) to 1 (fibers). for each endomicroscope position for control (blue) and RT (red).

Once the images were registered, using the pair of fluoroscopy images for each endomicroscope location, the location of the endomicroscope tip was pinpointed and the coordinates were mapped into the 3D CT volume (Fig. 5a–e). The coordinates of the location of the endomicroscope obtained between the coronal and sagittal views matched well with an average standard deviation of 0.67 *mm*. Using this registration method, each FE image (Fig. 5f) can be matched to each endomicroscope position on the CT (Fig. 5a–c). It is therefore possible to compare each CT image at each location (macro-imaging) to the corresponding FE image (micro-imaging).

### Comparison between Control and RT using CT and FE imaging

**Figure 6:**
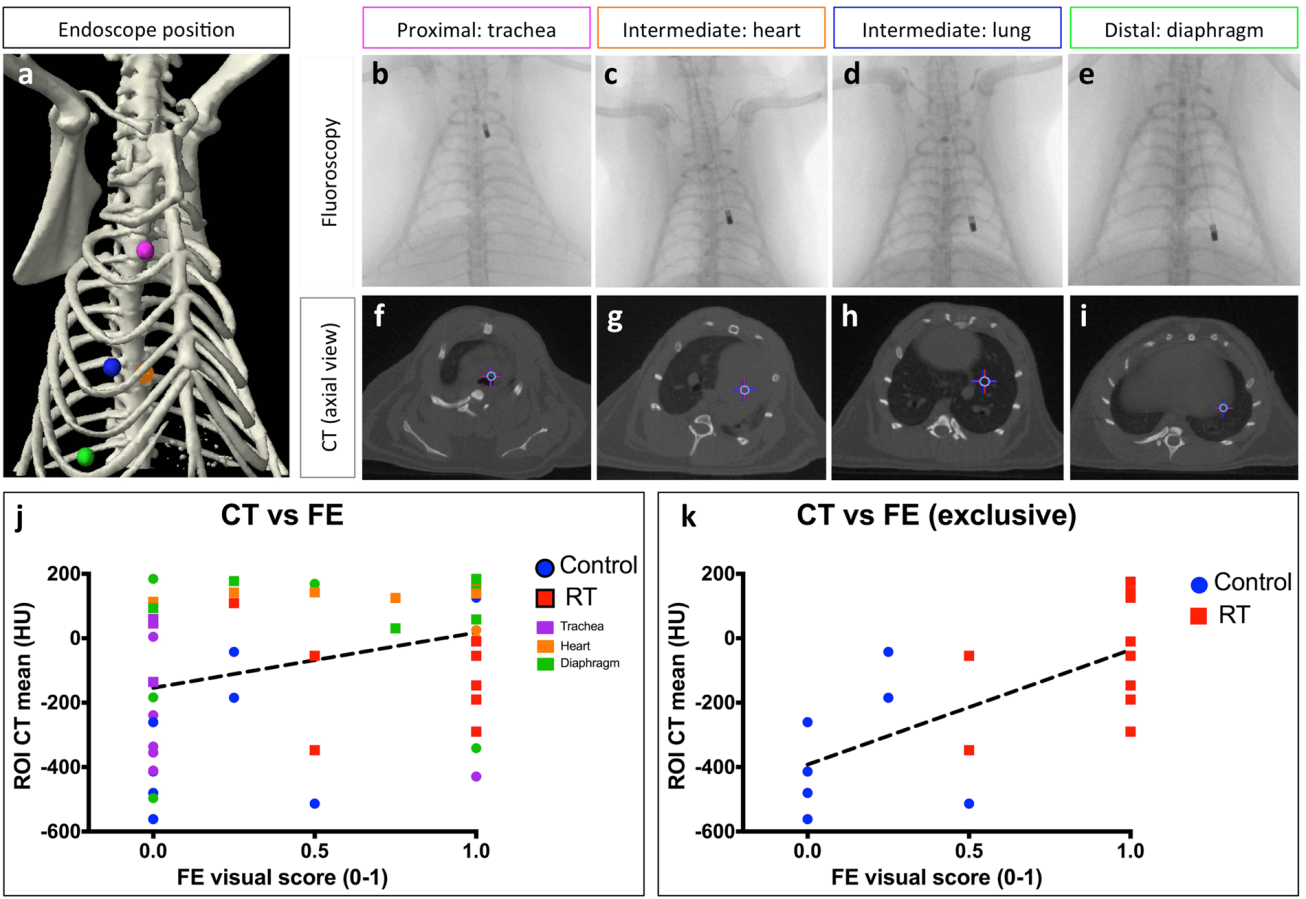
Correlation between CT and FE imaging at different endomicroscope positions. (**a**) 3D rendering of a rats’ ribcage from CT image showing 4 endomicroscope locations indicated by color-coded spherical ROIs. Proximal: trachea location is indicated in magenta with the endomicroscope location on fluoroscopy and CT image (**b** and **f**) respectively; Intermediate: heart location in orange (**c** and **g**); Intermediate: normal lung location in blue (**d** and **h**); and Distal: diaphragm location in green (**e** and **i**). (**j** and **k**) Correlation between CT value and FE score for each corresponding endomicroscope location. (**k**) Correlation excluding trachea, heart or diaphragm locations (only lung locations). Control: blue circles and RT: red squares. Dashed black line indicates linear regression.

The extent of RIPF in control and irradiated rats was evaluated and quantified with both CT and FE imaging. CT macro-imaging shows an increased lung density due to RIPF in RT (Fig. 5g–i) compared to control (Fig. 5a–c). The right lung appears dark in the normal control lung (less dense, air-like) but is gray (denser, tissue-like) in RT. This increase in density on CT images was quantified by computing the mean CT value (in Hounsfield units [HU]) of a spherical region-of-interest (ROI) of 3 *mm* in diameter around each endomicroscope position (Fig. 6a). HU are simply taken as is from the CT images which were calibrated on that machine for density to HU. Negative HU are normal for control lungs that are usually around −500 HU (air is −1000 HU) and fibrotic lungs are around 0 HU (similar to soft tissue or water). CT density increases on average but it is not homogeneous. The CT density quantification was taken as the average HU in a specific location ROI. Fibrosis appears as an overall density increase on CT images but with different fiber patterns and it appears in patches on histology.

Figure 5m shows a significant increase in CT numbers (lung density) in RT compared to control with a median of 111.3 *HU* and −184.8 HU, respectively (*p* = 0.0061).

FE images with the fluorescent collagen probe show increase fluorescence intensity and the presence of fiber structures in RT (Fig. 5l) compared to control (Fig. 5f). Figure 5n shows that there are significantly more fibers present on FE images of RT cases compared to control with a median of 1 and 0, respectively (*p* = 0.0025).

### Correlation between CT and FE imaging

Both CT and FE imaging were able to detect significant differences in lung fibrosis status between control and RT. To investigate this further, the correlation between CT and FE images at any given endomicroscope location was tested. Figure 6j shows significant correlation between CT values and FE scores for all the investigated endomicroscope locations yielding Spearman rank correlation *r* = 0.3423 (*p* = 0.015) and linear regression coefficient of determination *R* ^2^ = 0.1114.

However, some of the endomicroscope positions were considered to be more ambiguous and were classified into 3 categories: (1) proximal: trachea; (2) intermediate: heart; and (3) distal: diaphragm with examples shown in Fig. 6 (b and f), (c and g) and (e and i), respectively. (1) When the endomicroscope is located close to the trachea in the proximal airways, CT values might not accurately match the location on the fluoroscopy since the endomicroscope passes through the intubation and tracheotomy for FE imaging. As indicated on Fig. 6f the CT ROI at this location would comprise of a low density region (trachea) and a higher density region surrounding it, therefore giving a wide range of CT values. With respect to FE imaging in that proximal region, almost no fibers were observed (Fig. 6j, magenta). (2) Some positions show that the endomicroscope appears to be located in the heart region on CT images (Fig. 6g). Those events happened predominantly in the RT group (orange squares in Fig. 6j), as the fibrotic right lung tends to collapse, which shifts the mediastinum towards it, resulting in increased lung density in that region, that blends in with the heart region (Fig. 6g). (3) When the endomicroscope appears to be very distal and located in the diaphragm region (Fig. 6i), the CT values extracted from those locations are higher as the CT ROI encompasses tissue more than lung (Fig. 6j, green). Since the endomicroscope did not actually pierce the lung or touch the diaphragm, FE images show varying scores.

The correlation between CT and FE was also computed when excluding those ambiguous positions and keeping only the positions where the endomicroscope was located fully in the lung (Fig. 6h). Figure 6k shows the exclusive highly significant correlation between CT and FE at restricted endomicroscope locations with improved Spearman *r* = 0.643 (*p* = 0.0069) and improved *R* ^2^ = 0.4447. Control (blue circles) exhibit both lower CT values and FE scores than RT (red squares).

The challenge here is for small animal imaging since the size of the lung is a major constraint. We would apply these recommendations for small animal imaging to avoid being too proximal or too distal during FE image acquisition. Ideally, in the future, the CT would be acquired with the tracheotomy intubation in place and in the exact same position as for the FE and X-ray to avoid positioning errors to a higher extent. This would avoid mapping error back to CT that appear closer to the trachea but that are shifted due to the intubation.

### Comparison of collagen probe and immunofluorescence on *ex vivo* lung tissue sections

**Figure 7:**
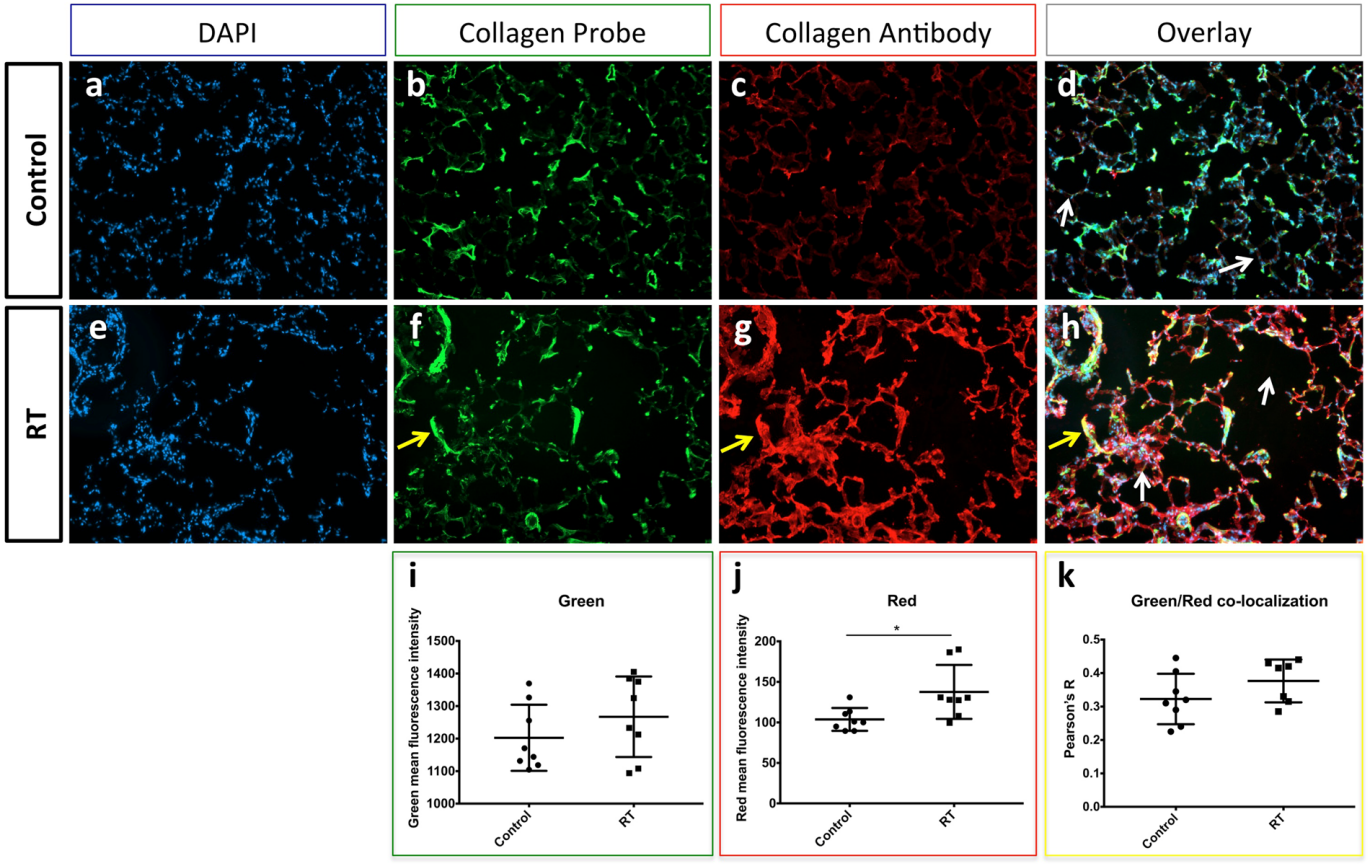
Microscopy images of DAPI (**a** and **e**, blue), collagen probe (**b** and **f**, green), immunofluorescence (**c** and **g**, red) and overlay (**d** and **h**) in *ex vivo* lung tissue sections comparing control (**a**–**d**) and RT (**e**–**h**). White arrows indicate normal alveolar structures in (**d**) and abnormal fibrotic regions with ruptured alveolar architecture in (**h**). (**i** and **j**) fluorescence intensity for the green or red channel, respectively, comparing Control (circles) and RT (squares). (**k**) Green and red co-localization Pearson’s R for Control and RT. Yellow arrows indicates a region of green (**f)** and red (**g**) co-localization that appears in yellow in the overlay image (**h**).

In order to validate the collagen probe further, it was tested on *ex vivo* lung tissue sections and compared to immunofluorescence using a collagen targeted antibody. Following *in vivo* imaging, lungs were harvested, frozen and sectioned for subsequent *ex vivo* microscopy analysis. Lung sections were stained with DAPI to highlight cell nuclei, with the collagen probe used previously *in vivo* and compared with immunofluorescence (antibody) of collagen (Fig. 7).

While the Control exhibits normal regular alveolar structures (Fig. 7d, white arrows), RT show a disrupted architecture with large empty regions and regions of dense fibrotic tissue (Fig. 7h, white arrows).

Furthermore, collagen immunofluorescence shows a significantly higher red fluorescence intensity (more collagen) in RT compared to Control (Fig. 7g and c, respectively), quantified in Fig. 7j (*p* = 0.03). However, this was not the case with the collagen probe (Fig. 7b,f and i) (*p* = 0.3823).

While the collagen probe might be lacking in sensitivity compared to the collagen antibody immunofluorescence, it appears that both co-localize well in the same regions of the lungs (Fig. 7f,g and h, yellow arrows) with an overall co-localization Pearson’s coefficient *R* = 0.35. No significant co-localization difference was observed between Control and RT (Fig. 7k) (*p* = 0.2319).

## Discussion

RIPF remains a major side effect of radiation therapy (RT) of lung and breast cancers. By combining prior CT imaging with fluoroscopy-guided fibrosis-targeted FE imaging, we were able to couple macro- to micro-imaging of RIPF in small animals. Thus, providing us with valuable location-specific information for clinical translation. This approach can inform clinician about the status of RIPF by relating collagen fibrosis content to increased lung density.

Hemithorax irradiation was chosen instead of full thorax in order for the rats to survive a dose of 18 Gy for 6 months, as the other lung can compensate breathing difficulties and can also serve as an internal control. RT side effects does indeed depend on the size of the irradiated volume and also localization (upper, middle or lower lobes). However, the goal was to have a lung fibrosis model, to actually measure fibrosis with different methods and treatments. Smaller volumes are indeed more clinically relevant, but might not be as useful to measure fibrosis or an overall effect of treatments. In addition, in a rat model where the lung measures about 2 cm by 1 cm by 1 cm it becomes very difficult to treat a smaller volume than the whole lung especially on clinical machines with MV photon beams. In the future we can imagine treating a small lung volume on a dedicated small animal irradiator but that requires more validation in order to use KV beams and still achieve comparable response to study fibrosis.

We synthesized and validated a new fluorescent collagen probe for FE imaging. It showed affinity to collagen and therefore fibrosis, both *in vitro* in a plate binding assay and *ex vivo* on lung tissue sections. The collagen probe did co-localize to the same regions as the antibody on *ex vivo* lung sections. However, the collagen probe was not as sensitive as the collagen binding antibody in immunofluorescence as it did not show a significant increase in fluorescence intensity in RT compared to Control. The *ex vivo*, quantification relied on fluorescence intensity whereas *in vivo* quantification was performed with scoring of fiber structures. We therefore focused on structural changes in fibrotic lung *in vivo* as opposed to simply averaging the fluorescence intensity. It is much harder to quantify similar fiber structures *ex vivo* considering that we are looking at a fixed tissue section. We determined *ex vivo* that the collagen probe binds to similar locations as the antibody since they showed agreement in co-localization. Furthermore, since, control lungs also contain collagen, the binding of the probe to control lung is not surprising. We can conclude that the collagen probe is not as sensitive as the antibody in terms of resolving different amounts of collagen present in the sample, but shows satisfactory binding patterns in terms of binding location. We were also able to observe fiber structures of increased fluorescence with *in vivo* FE imaging, that correlated with RIPF. We chose to use this fluorescent collagen probe for RIPF FE imaging, but other collagen or fibrosis specific probes could be devised instead. We also investigated the possibility of using a commercially available collagen probe: Col-F^19^. However, our *in vivo* FE imaging attempt using Col-F did not show fluorescent fiber structures in injured lungs. In any case, newly developed probes have to be validated *in vitro* and *in vivo* before usage with FE imaging.

The system used in this study is a confocal fluorescence endomicroscope (1.5 mm in diameter), and when used in small animals, the confocal probe is positioned directly in the airways in contact with the tissue of interest (“touch and see” strategy) and without bronchoscope channeling. It does not have white light capabilities, only fluorescence in the green and red channels, consequently, we cannot rely on bronchi images to guide the current probe design. Limited navigation options are available in small animal imaging, and there is no simple method to evaluate its location once in the body. Electromagnetic navigation is dedicated to human anatomy and not translatable to rat airways. Furthermore rat airways cannot accommodate any working channel equipped-fiber-optic device. With the proposed method we are capable of localizing the endomicroscope probe and gain more information on the fluorescence images since we now know where they originate from rather than relying on a whole lung estimate.

We visualized RIPF in a rat model with multimodal imaging requiring a micro-CT scanner, a C-arm and an endomicroscopy system. Performing fluoroscopy-guided FE imaging required that both the C-arm and the endomicroscopy system be in the same room with proper shielding for the x-rays and in close proximity to a surgery table to perform the required tracheotomy and probe injection. The CT scan could be acquired a day earlier and we were still able to position the rats in a similar manner to perform image registration between CT and fluoroscopy images. Access to all the equipment in the same location remains a challenge and requires prior planning and pilot studies to optimize the workflow when imaging multiple subjects.

In this study, we used a manual point matching method with an affine transform to register CT and fluoroscopy images based on anatomical landmarks. We were able to obtain good agreement for endomicroscope localization with a standard deviation of 0.67 *mm* in the z-direction, keeping in mind that the diameter of the endomicroscope probe itself is 1.5 *mm*. This method could be streamlined using automatic nonlinear registration methods^20^. Using fiducial markers visible on both CT and fluoroscopy images would make automatic registration easier and faster. Even though relying on anatomical landmarks might also be a source of error in the registration, these landmarks help quantify registration errors as they match directly with the specific anatomy of each individual rat.

Some endomicroscope positions were ambiguous as the endomicroscope tip appeared to be located in the trachea, the heart or the diaphragm. The endomicroscope remained in the airways but different factors might have influenced its location on CT images. Firstly, proximal locations appearing in the trachea were affected by the intubation and tracheotomy. Matching those locations back to the CT, where the rats were not intubated, could therefore be problematic as the anatomy of the proximal airways was disturbed. Secondly, the collapse of the irradiated lung, the shift of the mediastinum towards it and the increased lung density due to RIPF affected the CT contrast and the mapped location of the endomicroscope appearing to be located in the heart. Lastly, in some cases the endomicroscope was placed in a distal position and appearing to be located in the diaphragm when mapped onto the CT image. Again, the endomicroscope remained in the airways at all times and we did not observe piercing of the lung with the endomicroscope. However, respiratory motion of the diaphragm in distal regions of the lung is most probably the cause of these ambiguous locations. Image correlation between CT and FE was problematic at those endomicroscope locations and we obtained improved correlation when these were excluded from the analysis. Based on our experience in this study, we therefore recommend to keep the endomicroscope in intermediate locations to avoid the diaphragm by going too far or the trachea by remaining to close to the intubation.

We observed a correlation between lung density on CT images (macro-) and collagen fiber structures on FE images (micro-imaging) at any given corresponding endomicroscope location. The correlation improved when excluding ambiguous endomicroscope locations. Both imaging methods showed a significant increase in fibrosis in RT compared to Control. It is therefore possible to relate microscopic to macroscopic changes in lung tissue architecture.

In this study, we used fluoroscopy images to map the location of the endomicroscope back to the CT image. But, one could imagine going from an ROI on the CT, to placing the endomicroscope for FE imaging in that specific region with fluoroscopy guidance. This would require online and automatic image registration similar to image-guided surgery and would allow for live “optical biopsies” of targeted lung regions.

We developed and validated image-guided FE imaging for RIPF in small animals. This methodology can also be used for imaging other disease models such as idiopathic pulmonary fibrosis or any relevant model, provided one has a fluorescent probe for molecular imaging. One can then obtain both macro- and microscopic valuable information, providing a better understanding of the underlying molecular mechanisms of the disease *in vivo*.

## Methods

### Collagen probe and *in vitro* binding assay

The design of the collagen probe was based upon an MRI probe by Caravan *et al*.^16^ to image fibrosis *in vivo* in a mouse model^11^. The original MRI probe has a peptide structure identified by phage display with a demonstrated affinity for collagen. In order to use this probe design for fluorescence imaging, Gd-DTPA was replaced by fluorescein isothiocyanate (FITC) and since fluorescence is more sensitive than MRI, only one FITC molecule was incorporated. The collagen probe used in this study is as follows: Ac-Lys(Ac)-Trp-His-[*Cys-Thr-Thr-K(FITC)-Phe-Pro-His-His-Tyr-Cys]-Leu-Tyr-Bip-Amide.

We used an *in vitro* plate binding assay to test the affinity of the collagen probe to collagen (Fig. 1a). The wells of a 96-well plate were coated with rat tail collagen (RatCol® Rat Tail Type I Collagen, Advanced BioMatrix, USA). Collagen was diluted to a concentration of 1000 *μg*/*ml* in 0.1% acetic acid as described by the manufacturer and added to the wells (100 *μl* per well). Following an incubation of 1 hour at room temperature, the coated wells were washed with PBS. To avoid unspecific binding wells were blocked with 2% milk in PBS. The collagen probe was diluted in PBS to the desired concentrations (0, 1, 2 and 10 *μM*). Concentrations were verified by measuring the absorbance of the solution and calculating the concentration with optical density. The collagen probe was then added to the wells containing collagen or not (control) in 6 repeats wells and incubated for 30 minutes. The probe was then washed thoroughly with PBS. Following the binding assay, plate fluorescence was measured at 488 *nm* on a plate reader and the average fluorescence of 6 wells was computed for each concentration, with and without collagen coating.

### RIPF rat model

All experiments were approved by the Animal Care Committee at the Research Institute of the McGill University Health Centre and in accordance with the ethical guidelines of the Canadian Council on Animal Care.

RIPF was induced in rats as previously described^17^. Briefly, Sprague-Dawley female rats were placed in a prone position and imaged on a computed tomography (CT) simulation scanner under isoflurane anesthesia (Philips Brilliance Big Bore, Philips Medical Systems, Bothell, WA, USA) using an optimized small animal protocol (120 *kVp* X-ray tube voltage, 175 *mA* tube current, 0.37 *mm* in-plane resolution, 0.4 *mm* axial resolution). A hemithorax parallel-opposed 3D conformal treatment plan was designed (EclipseTMV 11.0, Varian Medical Systems, Palo Alto, California, USA) for each animal based on pre-treatment CT image. Animal positioning was verified with cone-beam CT prior to irradiation. A single dose of 18 *Gy* was delivered to the right lung with a 6 *MV* photon beam on a clinical Truebeam linear accelerator (Varian Medical Systems, Palo Alto, California, USA). Subsequently, fibrosis developed in the right lung for 24 weeks.

### CT imaging

Rats were imaged 24 weeks post-RT in supine position (on a Styrofoam holder for positioning reproducibility) under isoflurane anesthesia on a small animal CT scanner (X-RAD SmART, PXi, USA). Scanning protocol was optimized for rat lung imaging (100 *KVP*, X-Ray tube current: 1 *mA*, in-plane resolution: 0.2 *by* 0.2 *mm* and slice thickness: 0.2 *mm*).

### Fluoroscopy-guided FE imaging

Rats were anesthetized with intramuscular injection of ketamine/xylazine. A tracheotomy was performed for FE imaging as previously described^17^. Briefly, in supine position a midline cervical skin incision was done, and the cervical trachea was exposed by vertical separation of the muscles. A small incision between the tracheal rings was performed to pass a 14 G catheter through and allow the endomicroscope probe to pass.

Varying probe concentrations were tested in a bleomycin model based on experience from other fluorescent probe as well as *in vitro* data. The *in vivo* probe concentration in the bleomycin model was determined to have the best signal to background ratio. The collagen probe was injected intravenously via canulation of the jugular vein at a concentration of 10 *μmol*/*kg* in 1 *ml* of saline. 15 to 30 minutes following collagen probe injection, the rat was placed in supine position (as close as possible to the CT position on the Styrofoam holder) on the table of the C-arm. The endomicroscope was inserted into the airways, through the tracheotomy tube and placed at a certain location (proximal, intermediate or distal). FE images were acquired with a small animal endomicroscopy system (Cellvizio Dual band Lab, Mauna Kea Technologies, France) in the green channel (488 *nm*) for 10 seconds videos (at 9 frames per second). Immediately after FE imaging, two fluoroscopy x-ray images were acquired with a C-arm (BV Pulsera, Philips Medical Systems, USA) in coronal (from the top) and sagittal view (rotated 90 degrees). Then, the endomicroscope probe was moved to another location in the lung, FE images were acquired and another pair of x-ray images were taken. This was repeated for 4 to 5 locations (endomicroscope positions) per animal (Fig. 2).

### CT/fluoroscopy image registration

In order to obtain the endomicroscope location in 3-dimensions, each pair of 2D fluoroscopy images (coronal and sagittal view) were registered to the 3D CT image of the corresponding rat using the MINC Register software (http://www.bic.mni.mcgill.ca)^18^. Tags were placed on anatomical landmarks visible on both CT and fluoroscopy images such as sternum, vertebrae or ribs (Fig. 4). For sagittal view images, an average of 13 tags were used on the posterior region of each sternum bone junction (Fig. 4b and c) and on the anterior region of vertebrae junctions (Fig. 4a and b). Regarding coronal view images, tags were placed on the vertebrae at the point were ribs start to branch off (Fig. 4d and e) and on the most external region of the ribcage for each rib as it curves back (Fig. 4e and f). In most cases, 20 tags were used to register the coronal view images with points on each vertebrae and ribs, but in some cases, too many tags lead to big discrepancies in registration, therefore a limited number of tags (4 to 7) was used instead. This is most likely due to more uncertainty in tag placement on the ribs (with breathing motion), leading to mismatches between CT and fluoroscopy images. As the tags are placed and matched on both CT and fluoroscopy images, the corresponding transform was computed (3 translations, 3 rotations and 1 scaling) based on point-by-point matching of the tags.

The coronal view gave the x and z coordinates and the sagittal view gave the y and z coordinates (Fig. 4). Both z coordinates were matched with an average standard deviation of 0.67 *mm*. The average between the two zs was used as the z-coordinate. Once the 2D fluoroscopy images were registered to the 3D CT image (by applying the transform), the location of the endomicroscope was identified on the fluoroscopy image and its coordinates (x, y, z) were mapped back to its corresponding location on the CT image.

Positions that ended up outside the CT image were excluded from subsequent analysis.

### CT/FE image quantification at endomicroscope position

#### CT

Each endomicroscope position on the CT image was located and a spherical region of interest (ROI) with a 3 *mm* diameter was drawn (Eclipse V13, Varian Medical Systems, USA). Then, we computed the mean CT value in Hounsfield units (HU) for each ROI corresponding to each endomicroscope location.

#### FE

The fluorescent collagen probe highlights fibrosis, which appears as fiber structures on FE images. Each FE video was visually quantified for the presence (1), faint appearance (0.5) or absence (0) of fiber structures (Fig. 3). Scoring was performed by two independent observers and the average score was used.

### Collagen immunofluorescence and microscopy

Following FE imaging, rats were euthanized, the lungs were harvested and frozen in OCT. Lung tissue was then sectioned (10 *μm* thickness) on microscope slides for *ex vivo* evaluation. Slices were fixed in 4% formaldehyde for 15 minutes and washed in PBS three times. Blocking solution (10% goat serum, 0.3% Triton in PBS) was added to the slides for 1 hour and removed. Then, the primary antibody: rabbit anti-rat collagen type I (Cedarlane, Canada) was added (1:40 dilution in antibody dilution buffer: 5% goat serum, 0.3% Triton in PBS), incubated for 1 hour at 4 degrees and washed three times in PBS. The secondary antibody (dilution: 10 *μg*/*ml*): Alexa Fluor 647 goat anti-rabbit IgG (ThermoFisher, USA) was then incubated for 1 hour at room temperature and washed three times in PBS. Following the immunofluorescence procedure, the collagen probe (concentration: 10 *μM*) was added and incubated for 15 minutes then washed in PBS three times. DAPI (4′,6-Diamidino-2-Phenylindole, Dihydrochloride ThermoFisher Scientific, USA) was added (dilution: 1:1000) and washed for nuclei staining. Lung slices were then mounted and sealed with a coverslip. Slides were allowed to curate over night.

Microscopy images were acquired on a fluorescence microscope (AxioVert A1, Zeiss, Germany) using 10X magnification with DAPI, GFP (collagen probe) and mPlum (collagen antibody) filters. Two images per slides were obtained and fluorescence intensity was computed for both the green and red channels. Co-localization for green and red was performed with the Zen software (Zeiss, Germany) and the Pearson’s coefficient (r) was computed for each image.

### Statistical analysis

Statistical analysis was carried out using GraphPad Prism software. Non-parametric Mann-Whitney test was used to compare two conditions (control vs RT) and Spearman rank correlation was used to correlate two imaging acquisition methods (CT and FE). Differences and correlations were deemed significant when *p* < 0.05 and indicated with a star (*) on graphs.
